# Characteristics and Temperature Compensation of Non-Dispersive Infrared (NDIR) Alcohol Gas Sensors According to Incident Light Intensity

**DOI:** 10.3390/s18092911

**Published:** 2018-09-01

**Authors:** Humaira Hussain, JinHo Kim, SeungHwan Yi

**Affiliations:** 1 Department of Control & Instrumentation Engineering, Korea National University of Transportation (KNUT), 50 Daehakro, Chungjushi, Chungbuk 27469, Korea; humaira27@ut.ac.kr; 2 Department of Mechanical Engineering, Korea National University of Transportation (KNUT), 50 Daehakro, Chungjushi, Chungbuk 27469, Korea; wlsgh0614@naver.com

**Keywords:** ethanol gas, Non-Dispersive Infrared (NDIR), optical waveguide, dual-elliptical structure, temperature compensation

## Abstract

This paper discusses the output characteristics of the sensor response of infrared ethanol gas detectors based on incident radiation intensity. Sensors placed at each focal point of two elliptical waveguides were fabricated to yield two module combinations and to verify the output characteristics. A thin Parylene-C film was deposited onto the reflector surfaces of one module. The thermal properties were compared between the sensor (2.0 Ø) and sensor with a hollow disk (1.6 Ø), the disk being mounted at the end of one detector. The fabricated sensor modules were placed inside a gas chamber. The temperature was increased from 253 K to 333 K, over the concentration range from 0 to 500 ppm. As the temperature increases by 10 K, the output of sensor (2.0 Ø) without and with Parylene-C coating typically increased by 70 mV and 52 mV, respectively. However, the sensor output with the hollow disk showed an average decrement of 0.8 mV/50 ppm and 1 mV/50 ppm for module without and with Parylene-C deposition, respectively. For concentrations higher than 50 ppm, the estimation error was around ±5%. Further, the sensitivity to temperature variation and the absorbance of infrared (IR) reflection was found higher for Parylene-C coated module.

## 1. Introduction

Random breath testing and use of ignition interlock devices can reduce the risk of drunk driving and motor vehicle accidents by 20% [1] as the number of alcohol-impaired traffic fatalities has increased alarmingly [2]. Research has been conducted in several phases to observe human interaction with alcohol detection technology and also, the commercialization of alcohol detectors. The non-dispersive infrared (NDIR) gas detection method is advantageous over existing electrochemical sensors [3], metal oxide sensors [4,5] in the aspects of superior gas specificity, simpler fabrication, selectivity and quicker response time. Furthermore, periodic calibration is not required for NDIR sensors, unlike other proposed systems [6]. Any gas molecule under the influence of infrared (IR) light of intensity equivalent to a permissible vibrational transition, absorbs the light at a specific wavelength and suffers a transition between the different vibrational energy levels. The absorption strength determines the concentration of the target gas in a gas cell or chamber [7,8]. Infrared detectors with narrow band-pass filters respond to a very specific infrared spectrum for a particular gas. Even though prevalently used for CO_2_ [9,10], CH_4_ [11,12,13] and other gas detection [14], a few ethanol detectors pertaining to the same method have been reported so far [15,16,17,18]. The response of such sensors is affected by ambient temperature [14] implying that some temperature compensation method must be included in the sensor system. While the selectivity depends on the specific wavelength range for IR absorption, both sensitivity and detection limit can be enhanced by increasing IR intensity and optical length [14,19]. To achieve chemical, mechanical and electrical robustness [20,21], Parylene-C is widely used as a thin film coating [22,23,24]. In addition to securing chemical resistance and preventing vapor condensation on the surface of the reflectors, the sensor with the thin film coating was reported to show higher absorbance and reduced estimated gas concentration error, being significantly sensitive to temperature variation [25].

This research was conducted with the aim to build a compact, robust and stable NDIR alcohol detector acknowledging both the advantages and limitations of such an application. A unique dual-elliptical ethanol detector is designed in this research with two identical IR detectors, except one detector has an external hollow disk mounted on them because the performance of a sensor is extensively affected by optical waveguide design and intensity of light. Each of the detectors were placed on the focus of each ellipse while the source at the common focus. Afterwards, two similar modules were constructed but the reflector surfaces of one module utilize the protective properties of thin film Parylene-C coating and the results were justified. Then the temperature dependency was compensated, and finally this article verifies the measurement accuracy according to different light intensity incident on the two detectors.

## 2. Theoretical Background

### 2.1. Basic Structure and Principle of Non-Dispersive Infrared Gas Sensor

**Figure 1:**
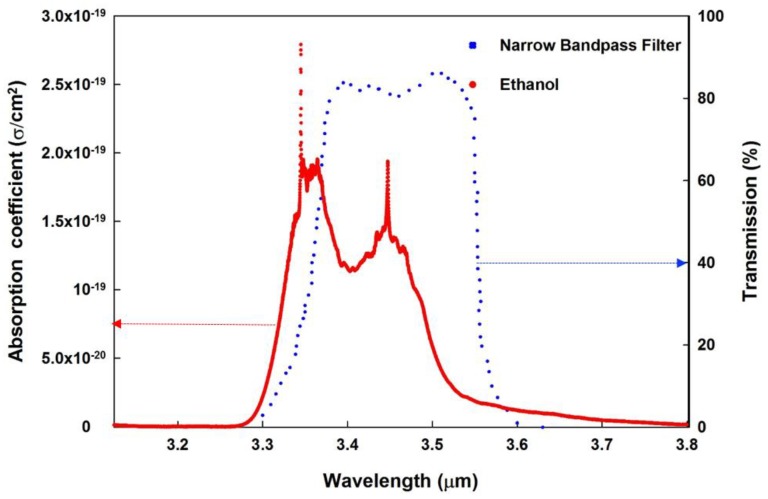
Superimposed transmission spectrum of the narrow band-pass filter on the absorption spectrum of ethanol gas.

The basic structure of an NDIR gas sensor system has been illustrated in the articles [19,26] and it comprises the following components: a broadband IR source along with its driving circuit, an optical waveguide, and an IR detector with a narrow band-pass filter attached to it. The operation of an NDIR gas sensor lies in the principle that when broadband radiation is emitted by an IR source, the narrow band-pass optical filter turns the IR detector into a much specific narrow band detector that screens out all the radiation except for a particular wavelength, which is absorbed by the sample gas. Typical absorption spectrum of ethanol is at 3.45 μm [27]. The IR absorption spectrum of ethanol and transmission spectrum of narrow band-pass filter which is used in this research, are plotted simultaneously in Figure 1. The overlapping of the two spectra significantly improves the detection of target gas at the mentioned peak wavelength.

The absorption of water is five times less than that of ethanol at 3.45 μm [28]. Also, the optical structure with Parylene-C coating has lower permeability to moisture and chemical resistance can be secured by preventing contamination and corrosion due to contact with air [21].

#### 2.1.1. Infrared Source

Regarding the selection of a light source (IR source), the intensity at the absorption wavelength of the molecule and the bandwidth of the light source are very vital. Ethanol gas has the highest absorption rate for infrared rays of 3.45 µm. Although theoretically ideal, it is not feasible to use an IR source emitting at a particular wavelength. Instead, a wideband blackbody infrared emitter is chosen. In the case of continuous wave light source for IR emission, the filament is heated to a high temperature to emit light, which is termed as the blackbody radiation theory. When a black body is the emission source, the total energy transmitted $I_{T}$ across all wavelengths, is in accordance with the Stefan–Boltzmann law [29,30,31]:

(1) $$I_{T}\propto \left(T_{\mathrm{body}}^{4}-T_{\mathrm{amb}}^{4}\right),$$

where $T_{\mathrm{body}}$ is the temperature of the black body, $T_{\mathrm{amb}}$ is the temperature of the surroundings.

Planck’s radiation theory describes the emission energy density function [31]. While the temperature of a blackbody emitter increases, the overall radiated energy is enhanced. Further, the peak of the radiation curve shifts to shorter wavelengths as a consequence of the increase in temperature. While Planck’s radiation theory yields the maximum emission of energy, the product of the peak wavelength and the temperature is constant [32].

The infrared light source (MILR17-900, developed by Intex, distributed by Importec, Polcenigo, Italy ) used in this research, continuously imitates the radiation pulses at a widely spectral range of 0 to 10 μm and the emission spectrum resembles that of blackbody. Table 1 lists the features of the IR source.

#### 2.1.2. Optical Waveguide

The intensity of light transmitted through an absorbing medium can be obtained by the Lambert-Beer law given in Equation (2) [33]:

(2) $$I_{d}{=I}_{0}\exp\left(-\alpha xl\right),\;I_{d}{=I}_{0}\exp\left(-\beta \left(T\right)x\right),$$

where $I_{0}$ is the initial incident light intensity, β(T) is the product of the gas absorption coefficient (α) and the optical path length (l) and *x* is the ethanol concentration (ppm).

This equation represents the co-dependence between optical irradiance and geometric parameters. The light intensity received at the detector can be determined by the gas concentration, optical path length and absorption coefficient of target gas, provided that the characteristics of the target gas have temperature dependent absorption properties. Hence, a larger optical length will enhance the sensitivity of the sensor at the same target gas concentration.

The characteristic absorption of a gas occurs in the analysis cell structure. Although larger optical path length is desired, the dimension of the chamber must be within acceptable range [34]. Several waveguide structure of various shapes and sizes have been studied over the years—cylindrical with circular cross section [26], spherical [9], combination of pairs of off-axis parabolic mirrors and right-angle mirrors [35] etc. Elliptical structures, in particular, can be characterized by the property that light traveling from one focus reaches the other focus. This shape was utilized for offering extended optical path length and multiple reflections of light from IR source to the detector end. A larger aperture combined within a smaller structure provides compactness [15,36]. Using dual wavelength differential principle, both symmetrical [12,37] and asymmetrical [38] single ellipsoid was optimized to achieve high sensitivity of the methane detector. Two intersecting symmetrical ellipsoid structure, similar to the proposed one in this paper were reported in [11,16]; source and detector are placed in focus of separate ellipse in [11] whereas the source is placed in a common focus and each detector at the remaining two focuses in [16].

The ethanol sensor structure presented in this paper can be termed unique due to the construction differences. This structure utilizes two identical ethanol detectors, each placed at the non-intersecting focus of the respective ellipsoid. This arrangement is implemented with one detector being intact at the upper part of the structure while the other is partially blocked by a hollow disk at the lower part which results in difference in received irradiation energies at the detector end. Hence, even though both the sensors take part in ethanol detection, the unblocked and partially blocked structures can be used to demonstrate the output characteristics of the sensors in case of different incident energy intensity. Both ellipsoids share a common focus where the IR source is placed while the detectors are on the non-intersecting focus. Moreover, thin film of hydrophobic Parylene-C is coated on the detectors of one of the two modules developed, in order to analyze and verify the improvement of electrical response along with preventing the reflector surfaces from decaying.

This design principle is based on the characteristics of the output of the sensor with varying temperature and concentration. The radiation energy intensity is proportional to the change in ambient temperature, so as the temperature is changed the output characteristic also change. The output voltage can be expressed as an exponential equation derived from Lambert–Beer’s law and are dependent on ethanol concentrations and temperature as given in Equation (3) [17]:

(3) VdF(T,x)=VoF(T)[exp(−β(T)x)],

where VdF(T,x) is the output voltage at a certain temperature and concentration of ethanol gas, VoF(T) is the output voltage at 0 ppm concentration.

In obtaining the parameters from regression analysis of voltage response curve using Equation (3), the concentration can be precisely estimated using the parameters in the following [16]:

(4) $$x_{\mathrm{ppm}}=-\frac{\ln(\frac{VdF}{VoF})}{\beta \left(T\right)}=-\frac{\ln\left(a\right)}{\beta \left(T\right)},$$

Here, ln(*a*) can be expanded in the form of a standard series and ethanol concentration can be estimated by substituting the series in Equation (4) [16]. Precise measurement and the trend of response of initial output voltage at 0 ppm concentration, β(T) and the output voltage curves after injection of ethanol at different ppm levels over a varying temperature range will determine the accuracy of measurement.

#### 2.1.3. Infrared Detector

The energy transmitted by an IR source is received at the IR detector. The detector used in our analysis is a thermopile detector. When the thermopile detector is integrated with an appropriate band-pass filter, it can measure certain gases such as CO_2_, CO, CH_4_, ethanol etc. The amount of light received by the detector is converted to the output voltage depending on the temperature difference between the high and low temperature contacts. The output voltage of the thermopile detector is governed by Equation (5) [39]:

(5) $$V=\int_{T_{\mathrm{Low}}}^{T_{\mathrm{High}}}\Delta \alpha_{s}dT,$$

where $T_{\mathrm{High}}$ is the temperature of the high-temperature contact, $T_{\mathrm{Low}}$ is the temperature of the low-temperature contact, and $\alpha_{s}$ is the Seebeck constant.

The HIS A21E3.45G5600s manufactured by HEIMANN Sensors^©^ GmbH (Dresden, Germany) used in this experimental study can operate at a temperature range of −40 to 120 °C and offers an integrated linear temperature sensor output of 15 mV/°C sensitivity. The detector is attached with narrow band-pass filter centered at 3.45 μm for precise ethanol gas detection. To overcome noise interference from external amplification circuit in lower gas concentration, thermopile detectors are often integrated with Application Specific Integrated Circuit (ASIC) chip. This reduces noise component and enhances the output properties of thermopile.

#### 2.1.4. Geometric Realization of an Elliptical Cell

Each of the ellipsoid structure is a symmetrical and identical. The semi-major and semi-minor axis of the geometrical shape can be denoted by a and b. The IR source is placed at one focal point at a distance $c=\sqrt{a^{2}-b^{2}}$ and the detector is placed at the other focal point; at a distance 2*c* from the source, on the major axis. The surface area of the detector area is S and K is the reflectivity of the structure [37]. The radiant intensity from the source incident at a certain angle I(θ) and radiant flux received at the detector ϕ end can be described by the following equations [37]:

(6) $$I\left(\theta \right)=I_{a}\cos\theta ,$$

(7) $${\phi =I}_{a}\frac{S}{{\left(2c\right)}^{2}}e^{-\alpha (2c)}{+2\pi I}_{a}K\int_{\theta_{1}}^{\theta_{2}}e^{-\alpha l}\cos^{2}\theta \mathrm{dcos}\theta ,$$

where $I_{a}$ is the radiant along the axes.

According to Equation (7), the first part in the equation corresponds to flux received without any reflection while the second part corresponds to the flux received after reflection [38]. From this equation, it can be assumed that if the detector area is blocked by any external means, received flux is altered.

## 3. Sensor Module Fabrication and Analysis

With the aim of finding the optimum combination to compensate for the effect of different energy densities and sensitivity to ambient temperature variation, two sensor modules were constructed. A thin film of hydrophobic Parylene-C was deposited on the reflectors of one of the sensor modules, as a preventive measure for water condensation and chemical corrosion. To verify the output characteristics of the infrared detector according to the amount of incident light, a hollow disk was mounted in front of one of the two infrared detectors present in the waveguide structure—having a constant optical path length in both the sensor modules. One detector without any hollow disk (2.0 Ø) is placed in the upper part, while another equipped a hollow disk of 6 cm (1.6 Ø) was placed in the lower part of the sensor structure [17]. The hollow disk was mounted to examine the amount of incident light on the infrared detector according to IR intensity. Upon completion, the module was used in the experiment to study the characteristics of the ethanol gas sensor at various temperatures and concentrations.

### 3.1. Sensor Module Design Considerations and 3D Modelling

**Figure 2:**
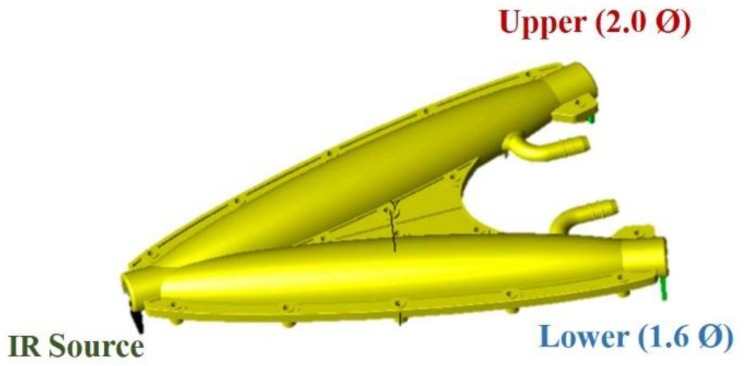
3-D modeling of the sensor module.

Even though the proposed waveguide has two identical symmetrical ellipsoids, the separation between the major axis affects the light reaching the detector. The modeling and optimization of the cell shape has already been analyzed in [40]. The optimum separation angle between the two major axis is found to be 30 degree. This angle was chosen as a tradeoff between maximum irradiance and incident ray count. Maximum irradiance also obeys the condition that the IR source is placed at the focal point. Ray-tracing simulations showed multiple reflections along the path length which ensures larger output voltage and improved sensitivity. The length of the major and minor axis of each ellipse is 154.95 mm and 20 mm. During the initial simulation, the light is transmitted with a source power 600 mW, the number of incident rays were 100,000 and the reflectivity of the elliptical surface is 95%. The 3-D modeling of the fabricated sensor module is exhibited in Figure 2. The IR source was placed on the common focus of the dual elliptical waveguide while the detectors with no disk (2.0 Ø) and with disk (1.6 Ø) were modeled to be on the remaining focal point of each ellipse.

**Figure 3:**
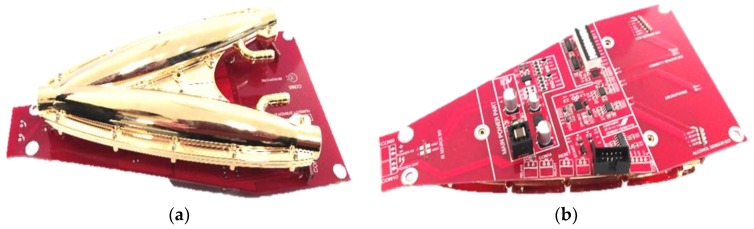
(**a**) Fabricated dual elliptical ethanol sensor module; (**b**) Printed circuit board below the dual elliptical structure.

The 3-D shape was designed using Solid-works^®^ (Dassault Systèmes, Vélizy-Villacoublay, France) and the simulation was performed using Trace-Pro^®^ (Lambda Research Corporation, Littleton, CO, USA). The model was later fabricated in a highly precise molding structure to build the optical waveguide with functional plastic. The fabricated dual elliptical structure with the source and detectors assembled onto it is mounted on a printed circuit board shown in Figure 3a,b. The circuitry was placed separately from the waveguide in order to keep the signal to noise ratio unaffected.

### 3.2. Signal Conditioning Circuit

**Figure 4:**
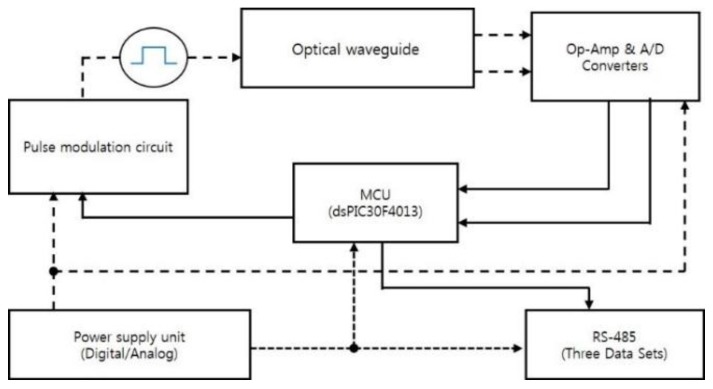
Block diagram of signal conditioning circuit.

For signal conditioning, the two electrical power supply-analog and digital are kept apart to acquire the stability. This is because the IR source is modulated at a high input power of 600 mW. A variable resistor is used to adjust the peak analog supply voltage of 9 V applied to the IR source. The on- and off-time pulses are controlled by the MicroController Unit (MCU). The integrated IR detectors with ASIC chip were installed. A pre-amplifier is housed in the same packaging. The detector signals are enlarged by a pre-amplifier (MAX4239, Maxim Integrated Products Inc., San Jose, CA, USA) which offers low drift and ultra-low offset. The analog signals are converted into digital ones by an A/D converter after the signals have passed through two stages of differential OP-AMP. This amplifier helps to measure the difference between the output voltages more precisely and improving the sensitivity. Output signals are transmitted through the RS-485 (Analog Devices, Inc., Norwood, MA, United States) chip to the computer for further analysis. The block diagram of the circuitry is presented in Figure 4. The analog signals are represented as dotted lines whereas solid lines imply digital signals.

### 3.3. Simulation Result

**Figure 5:**
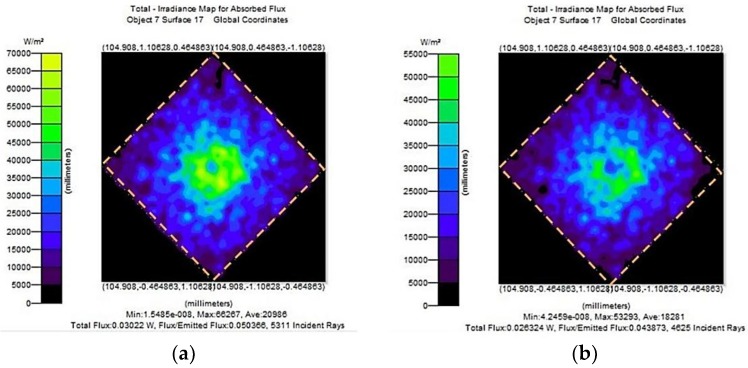
Initial simulation results (**a**) normal structure (2.0 Ø); (**b**) with hollow disk (1.6 Ø).

The infrared active region of 1.2 mm × 1.2 mm was formed at the center of the infrared sensor shown in Figure 5, and the simulation result was obtained assuming that the active region absorbs 100% of the incident light. Reflecting on the initial conditions, the simulated results of the incident energy absorbed by different waveguide structures are shown in Figure 5a,b. In Figure 5b, the amount of incident light decreases as the size of the hole in the disk decreases. This phenomenon can be explained by Equation (7). When the hollow disk is mounted before the detector, the angular range of incident light emission *θ* becomes limited by the change in the area of incidence. Consequently, the received energy at the detector will be less than that of intact detectors. In the case of an infrared detector (2.0 Ø) without a hollow disk, the radiant power per unit area at the detector was observed to be 0.030 W, whereas for a detector with a hollow disk (1.6 Ø) the power simulated was 0.026 W.

## 4. Experimental Method

**Figure 6:**
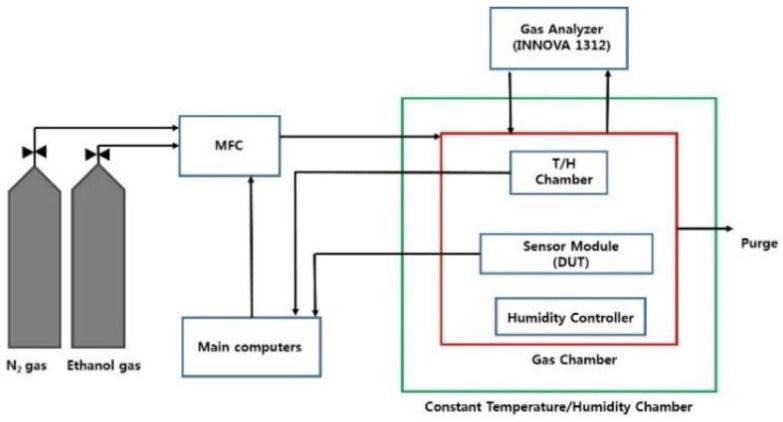
Block diagram of experimental setup. MFC: Mass Flow Controller; DUT: Device Under Test.

To evaluate the output characteristics of the sensor module according to the temperature and the ethanol gas concentration, a measurement system as shown in Figure 6 was set up. First, the sensor module was inserted into a closed gas chamber inside the thermo-hygrostat, and the temperature was adjusted from 253 K to 333 K at step intervals of 10 K except at the temperature at 298 K. After adjusting the temperature, more than 4 h had passed before the thermal equilibrium state of the sensor module was attained. When the variation of the output of the temperature sensor was within ±5 mV of the previous experimental result, the mass flow controller (MFC) of gas was turned on. Subsequently, the gas inside the chamber was purged with high-purity nitrogen (99.9999%, class 6) to reach 0 ppm by waiting for 1 h or more. The experiments were then carried out by feeding a high concentration of ethanol gas through the mass flow controller of gas into the sealed gas chamber, from 0 to 500 ppm in 50 ppm step units.

A multi-gas analyzer (INNOVA-1312, LumaSense Technologies A/S, Ballerup, Denmark) was used to standardize the successive measurements of ethanol concentration with a precision of 1 ppm or less. The gas analyzer is calibrated and attached with special filters for accurate ethanol measurement. The connection from the analyzer to the gas chamber is properly insulated. The output of the sensor module according to the concentration was recorded as a voltage in the computer, and the corresponding experimental data was saved using the RS-485 communication.

## 5. Result & Discussion

### 5.1. On-Off and Response Time

**Figure 7:**
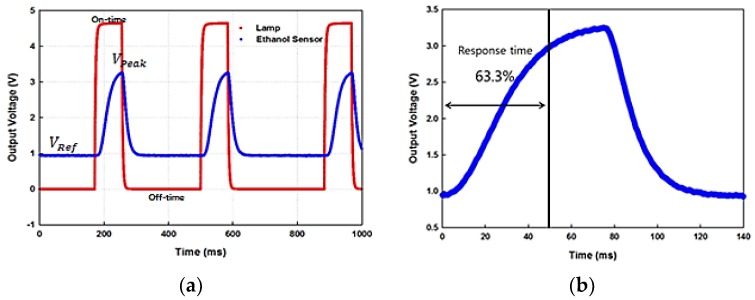
(**a**) On-off time of the IR source and response of the sensor; (**b**) response time of the sensor.

The IR source is modulated for pulse generation and recorded on-off time is shown in Figure 7a. The optimum on-off time of IR source is found to be 100 ms and 300 ms, respectively. The values were chosen after iterative experiments. It takes a while for the source to transmit IR energy to the detector, so that the sensor response can reach to the peak amplitude level but even if the on-time is further extended, the peak voltage does not increase significantly. On the other hand, if the off time is shorter, peak value of voltage cannot be reached. In case of longer off time, the data sampling duration will be higher. The response time of the ethanol sensor is observed to be 48 ms as seen in Figure 7b.

### 5.2. Output in Absence of Target Gas

**Figure 8:**
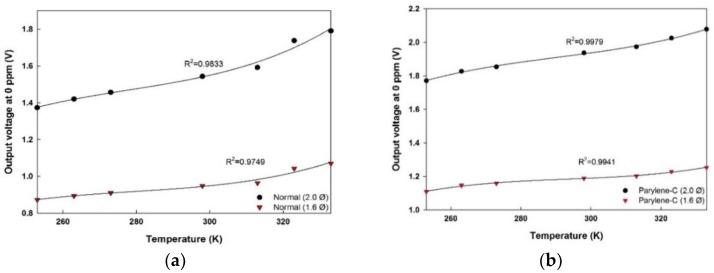
Output voltages at 0 ppm ethanol concentration with varying temperature obtained from (**a**) normal sensor module; (**b**) sensor module with Parylene-C deposition.

When the gas chamber was purged with N_2_ gas to obtain 0 ppm concentration of target gas, the output voltages were also recorded and downloaded. The signal values at different temperature for each detector of each module was plotted in Figure 8. It is evident that even in the absence of target gas the outputs are different. The initial output voltage varies because of the constructional differences between the sensor modules. These differences among the results can again be explained by Equation (7). As the temperature rises from 253 K to 333 K at 10 K interval (including the interval between 273 K and 298 K), the initial output voltage of the (2.0 Ø) sensor with no Parylene-C increases by 70 mV on average. With the hollow disk being mounted (1.6 Ø), the same sensor module output rises by 33 mV, as a result of change in light transmitting angle with respect to the major axis of elliptical waveguide reaching the detector end. The Parylene-C deposited sensor module with structure (2.0 Ø) increases by 52 mV with each ascending temperature setting. However, at the output of the detector with a hollow disk, we observed that the initial output voltage increases by 24 mV on average with the increase in temperature. The increment in voltage showed a temperature dependency, resulting in a coefficient of determination, R^2^ = 0.9833. The linearity quantifies the output variation with respect to IR source power. Conversely, the prototype with Parylene-C deposition showed linear temperature-dependent characteristics with a higher coefficient of determination, R^2^ = 0.9975 along with higher output voltage. The output voltages at 0 ppm for each module are presented in Figure 8a,b.

The output voltages increase as the ambient temperature increases from 253 K. This can be characterized by Stefan-Boltzmann law in Equation (1). As the ambient temperature rises higher, the temperature of the source also increases resulting in a greater difference between the source and ambient temperature. Consequently, according to Equation (1), transmitted light intensity improves proportionally to the difference between the fourth powers of the two temperature. The output voltage at the detector end is also a measure of incident light intensity. Hence the output voltages enhance with the increase in ambient temperature conditions.

The β(T), the product of the gas absorption coefficient and optical path length can be determined by the regression analysis of the second-order polynomial function of measured temperature. The values of the initial voltage and gas absorption coefficient recorded at each temperature for both modules are given in Table 2.

Table 2 shows that the product of absorption coefficient and optical path length doesn’t show much temperature dependency. However, the values of β(T) are stored and are formulated as a second order polynomial function of the temperature setting.

### 5.3. Temperature Dependency

The output voltages received over the range of concentration used in this experiment for each temperature setting are stored and plotted. The signal curves follow temperature fitting according to Equation (5) depending on the concentration value. When the ethanol gas is injected inside the chamber, the absorption of the target gas in the optical waveguide under the influence of IR light is initiated around 3.45 μm. Due to the exponential relationship between the output voltage and concentration corresponding to Equation (5), the sensor output decreases as the amount of ethanol is increased. Also, as described in the previous section, the increase in ambient temperature yields in the improved radiation energy intensity. The output voltages increase at higher temperature for the same ethanol concentration injected in the gas chamber but decay with increased gas absorption at the measured temperature.

**Figure 9:**
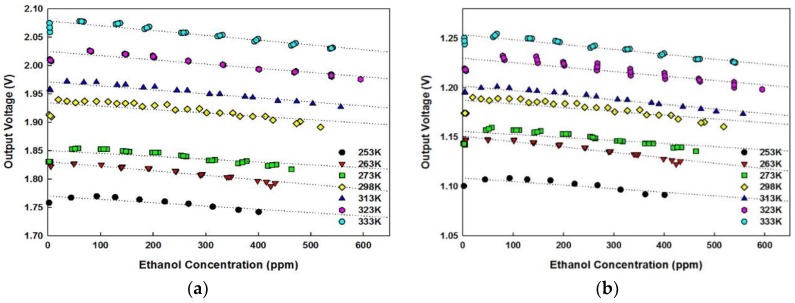
Parylene-C sensor output voltages of (**a**) detector with no disk (2.0 Ø); (**b**) detector with hollow disk (1.6 Ø).

The experimental results show that, with a hollow disk attached, when the concentration is higher than 10 ppm, the output of the sensor without thin film decreases by 0.8 mV/50 ppm interval on average, over the range of ethanol concentration tested in this experiment. However, the output voltage of the sensor module with deposition decreases by 1 mV/50 ppm interval on average for concentrations above 10 ppm. Compared to the module with no thin film deposition, Parylene-C module shows improved voltage response and higher sensitivity to varying concentration and also to temperature change since it can minimize the effect of infrared scattering caused by adhesion of water vapor. The output voltages of Parylene-C deposited module are presented in Figure 9a,b.

Therefore, along with the voltage contribution from IR source which is independent of the ethanol gas absorption but depends on ambient temperature, system response is also affected by absorption of ethanol around 3.45 μm with respect to varying temperature and concentration.

### 5.4. Temperature Compensation

**Figure 10:**
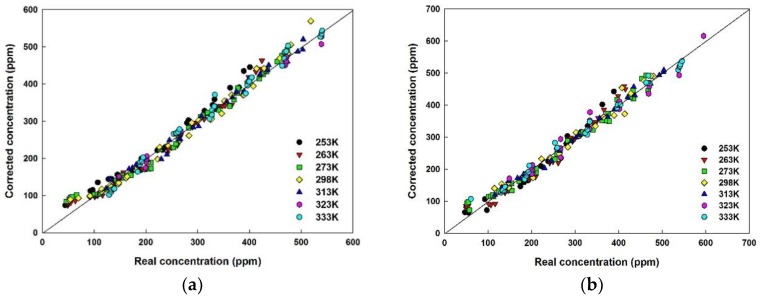
Corrected concentration of Parylene-C module at (**a**) detector end with no disk (2.0 Ø); (**b**) detector end with hollow disk (1.6 Ø).

The ethanol concentration can be evaluated by using the expanded Equation (4). The initial output voltages at 0 ppm concentration show a high dependency on temperature. The average voltage ratios calculated at the measured temperature is constant over the entire concentration range. The ratios are implemented using 3rd order polynomial equations with varying ambient temperature. Again, since the denominator of the Equation (4) is almost constant at a certain temperature in the range in which the experiments are conducted, it is programmed in the MCU as a second order polynomial of ambient temperature. The estimated concentration is solely temperature reliant and can be measured accurately. The temperature compensation algorithm was formulated [10] and ethanol concentration was estimated at each measured temperature. Despite the temperature compensation, an offset from the temperature-compensated concentration occurs from the real concentration due to the energy received by the band-pass filter at wavelengths other than at 3.45 μm. Since the received thermal energy of an IR detector is directly proportional to the output voltage, the response is always observed with an offset, which depends on the thermal properties but is independent of the variation in the ethanol concentration. The difference between the actual concentration and the estimated concentration was obtained in the entire concentration range, and the average of the difference values was used to derive the correction value for every temperature point. These values were adjusted in the MCU algorithm to obtain accurate ethanol concentration. The corrected concentration is plotted in Figure 10a,b.

As evident from Figure 10, the concentration measured at both detector ends are almost similar. The corrected concentration shows reliable linearity corresponding to actual concentration in the gas cylinder which asserts that the temperature compensation algorithm has been successfully implemented. Although the received light intensity and subsequent output voltages of one detector are different from another, the ratio of the output voltages ($\frac{VdF}{VoF}$) at the measured temperature is analogous for the entire concentration range. Therefore, precise estimation of ethanol concentration can be achieved irrespective of the IR intensity received by the detector. The consistent ratio of output voltages across the range of concentration ensures the stability of the sensor module.

### 5.5. Error and Detection Limit

The experiment was conducted for several months and the error in concentration was recorded. The error percentage for 50 ppm concentration was much higher i.e., an error range starting from 10% or greater for both the sensors with and without deposition at a temperature less than or equal to 298 K was achieved.

**Figure 11:**
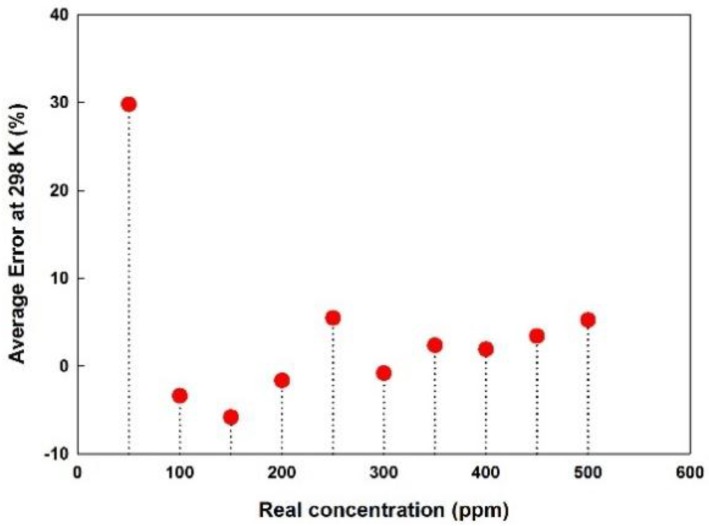
Average error of concentration measurement of Parylene-C module at 298 K.

Differences between initial calculations and regression analysis yielded higher error below 50 ppm concentration. However, in the case of more than 50 ppm, the average error range is around ±5%. Although the error percentage is quite similar between the sensor with deposition and without deposition, since the sensor module deposited with Parylene-C has a larger output voltage change depending on the gas concentration than the normal sensor module, a more accurate estimated concentration can be obtained. The average error between each combination of the detector at 298 K for Parylene-C deposited module are plotted in Figure 11.

## 6. Comparison and Conclusions

The ethanol detector has been modeled and monitored for over two years and the progress is summarized in Table 3. While modeling using a reference detector sensor module of a higher accuracy can be achieved over entire concentration range, subsequent research work emphasized on verifying the sensor’s response according to different incident ray intensities using two identical detectors at 3.45 μm. This has been established by partially blocking the detector i.e., incoming light intensity. Temperature compensation algorithm was implemented. The higher sensitivity and higher absorbance were obtained by using the properties of Parylene-C coating. However, the geometrical shape of the waveguide structure is the same for all the mentioned works in Table 3. In this paper, the temperature characteristics, temperature compensation, and voltage output correction method were described according to the amount of incident light on an infrared sensor. The analysis from simulation allowed us to predict the amount of light entering the infrared sensor located at each focal point, and the output voltage was verified through experiments. The temperature dependent parameters were obtained to estimate ethanol concentration accurately irrespective of received IR light. It was confirmed that the mean error between the actual gas concentration and measured concentration after temperature compensation and calibration was typically ±5%, suggesting the applicability as a sensor module for alcohol detection.

## Figures and Tables

**Table 1:** Features of infrared (IR) source (MILR17-900).

Parameter	Typical Value
Spectral output range	1–10 μm
Emitter Surface Area	1.7 × 1.7 mm^2^
Working temperature	750 °C
Power consumption	980 mW
Modulation frequency	0–100 Hz (optimized at 2.5 Hz)
Warm up time	<30 ms
Lifetime	>5000 h at 750 °C

**Table 2:** Values of initial voltage and gas absorption coefficients at various temperatures.

Temp. (K)	Normal (2.00 Ø)		Parylene-C (2.00 Ø)	
	V_oF_ (T)	β(T)	V_oF_ (T)	β(T)
253	1.3736	5.9 × 10^−5^	1.7712	5.0 × 10^−5^
263	1.4204	5.6 × 10^−5^	1.8271	5.1 × 10^−5^
273	1.4572	5.1 × 10^−5^	1.8536	4.8 × 10^−5^
298	1.5435	5.4 × 10^−5^	1.9375	5.0 × 10^−5^
313	1.5920	5.3 × 10^−5^	1.9731	4.8 × 10^−5^
323	1.7378	5.1 × 10^−5^	2.0255	4.8 × 10^−5^
333	1.7909	5.3 × 10^−5^	2.0784	5.1 × 10^−5^

**Table 3:** Comparison of design principle and performances among this sensor and previously reported ethanol sensors [16,17,41].

Design Principle	Error (%)	
One ethanol and one reference detector	±5%	[16]
One ellipsoid partially covered with tape	20–25% at <200 ppm −10 to 1% at >200 ppm	[41]
Hollow disk (1.6 Ø) inserted before one detector	±15% at <100 ppm −1.2–3% at >100 ppm	[17]
Hollow disk (1.6 Ø) inserted before one sensor, detectors of one of the modules coated with Parylene-C	More than 10% for ≤50 ppm ±5% at >50 ppm	This
